# Data and analyses of woody restoration planting survival and growth as a function of wild ungulate herbivory

**DOI:** 10.1016/j.dib.2017.07.002

**Published:** 2017-07-08

**Authors:** Joshua P. Averett, Michael J. Wisdom, Bridgett J. Naylor, Mary M. Rowland, Bryan A. Endress

**Affiliations:** a Eastern Oregon Agriculture and Natural Resource Program, Oregon State University, Badgley Hall 205, One University Blvd., La Grande, OR 97850, USA; b USDA Forest Service, Pacific Northwest Research Station, 1401 Gekeler Ln., La Grande, OR 97850, USA

## Abstract

These data and analyses support the research article “Wild ungulate herbivory suppresses deciduous woody plant establishment following salmonid stream restoration” Averett et al. (2017) [1]. The data and analyses presented here include: (1) planting density, survival and growth (two years post restoration) of riparian plantings along an ~11 km stream reach in northeastern Oregon as a function of herbivory treatment (protected/not protected from wild ungulate herbivory), habitat type, and planting species; and (2) abundance and height distributions of naturally occurring deciduous woody species along the restored stream reach two years post restoration. Survival and growth analyses are provided as output from multiple logistic and mixed effect regression models respectively.

**Specifications Table**

**Table:** 

Subject area	*Biology*
More specific subject area	*Riparian vegetation restoration and herbivory*
Type of data	*Figures and Tables*
How data was acquired	*Repeat vegetation sampling along permanent riparian transects.*
Data format	*Analyzed, Raw*
Experimental factors	*Woody species were planted along an 11-km stream reach. The plantings were exposed to two herbivory treatments (1) protected; and (2) unprotected from elk (Cervus elaphus) and mule deer (Odocoileus hemionus) herbivory. Planting occurred in wet and dry meadow habitats.*
Experimental features	*Planting growth and survival were measured at the end of two growing seasons. Planting densities were determined at the end of the first growing season, and naturally occurring deciduous woody species abundance and height was measured two years post-restoration.*
Data source location	*Data was collected at the Starkey Experimental Forest and Range of the USDA Forest Service in northeastern Oregon, USA. (45˚12′ N, 118˚ 3′ W)*
Data accessibility	*The data are available with this article and within*Supplementary files.

**Value of the data**

• These data present survival and growth of woody riparian restoration plantings across two levels of wild ungulate herbivory and can be compared to other restoration planting studies.
• These data provide post-restoration abundance and heights of naturally occurring deciduous woody species that can be compared to riparian vegetation recovery in other studies.
• Future repeated measurements can be compared to this data to reveal long-term relationships between wild ungulate herbivory and woody riparian vegetation development.
• These data allow researchers to extend the analyses.

## 1 Data

**Fig. 1:**
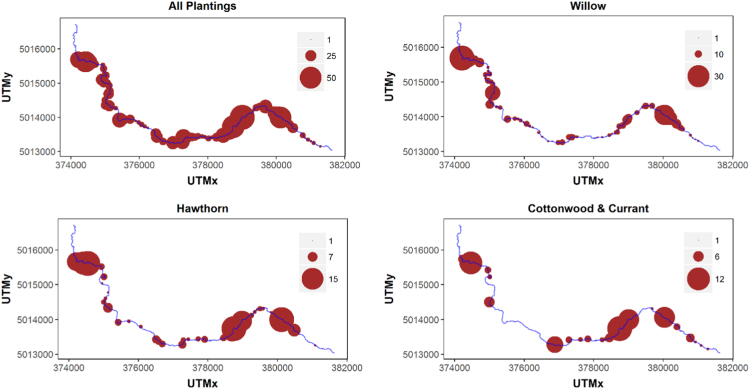
Sampling density for all riparian restoration planting species combined and for the four species – *Crataegus douglasii* (hawthorn), *Populus balsamifera* (cottonwood), *Ribes aureum* (currant), and *Salix spp.* (willow) – sampled for growth and survival analyses along the Meadow Creek restoration reach in northeastern Oregon. Larger symbols (red circles) indicate higher density of plantings. The blue line depicts the Meadow Creek stream channel.

**Fig. 2:**
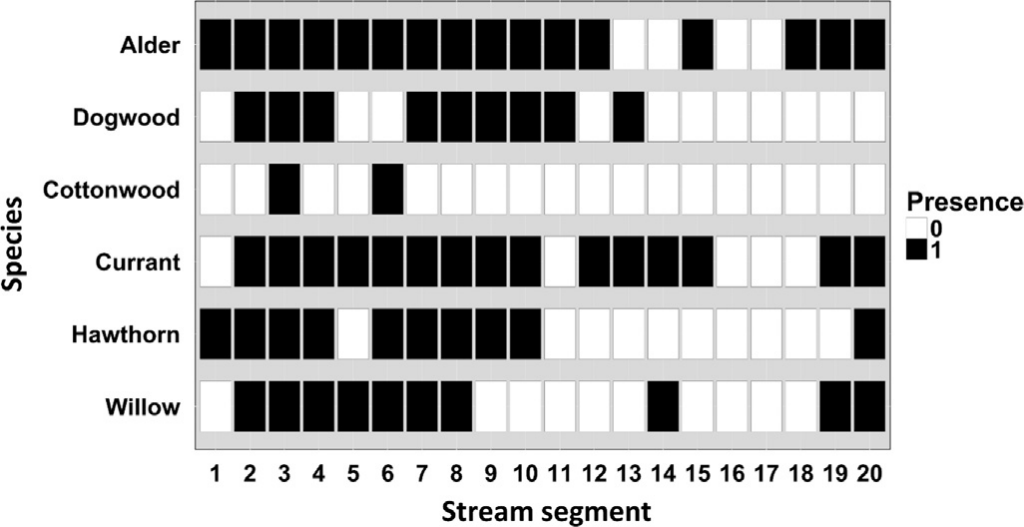
Presence (1) and absence (0) of naturally occurring deciduous woody species within ~0.5-km increments along the restored Meadow Creek stream reach two years post-restoration. Stream segment number increases from east to west along the restored stream reach.

**Fig. 3:**
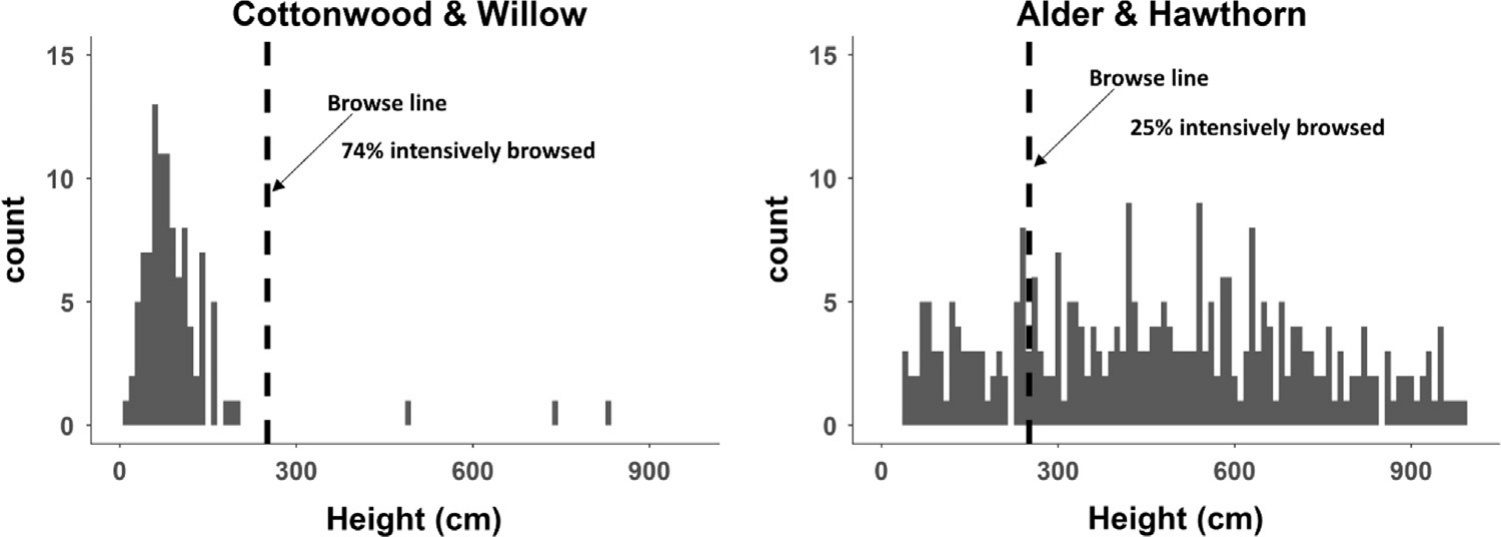
Height distributions for naturally occurring deciduous woody species that are highly (cottonwood and willow) and less (alder and hawthorn) preferred by elk and deer within the Meadow Creek riparian area in northeastern Oregon. The dashed lines show the browse line, above which, plants escape heavy browsing pressure by wild ungulates in this system [1]. Percentages of intensively browsed plants that were available to heavy browsing pressure (≥50% of photosynthetic area below 2.5 m) are shown for both categories.

The presented data was obtained by sampling riparian vegetation along 191 permanently located riparian transects, and by tracking the growth and survival of 1057 woody riparian restoration plantings exposed to two different herbivory treatments (protected/unprotected from wild ungulate herbivory) for two years following restoration, and include: (1) sampling densities of plantings by species along the restored reach (Fig. 1); (2) survival analyses using multiple logistic and mixed effects logistic regression (Table 1, Table 2); (3) growth analyses using mixed effects regression (Table 3, Table 4); (4) abundance (presence/absence) of naturally occurring deciduous woody species along the restored stream reach two years following restoration (Fig. 2); and (5) height distributions of naturally occurring deciduous woody species for highly and less preferred (by elk and deer) species as well as percentage of individuals within those two categories subjected to intensive browsing pressure (Fig. 3). Corresponding datasets are provided within a Supplementary file to this paper. Refer to [1] for detailed interpretation and discussion.

**Table 1:** Likelihood ratio tests of significance for main effects and two-way interactions from survival analyses using multiple logistic and mixed effects logistic regression analyses. Site (groupings of transects for each ~1-km increment along the restored stream reach) was used as a random effect in the mixed effect model.

Likelihood ratio tests of significance for main/interaction effects						
	Multiple logistic regression			Mixed effects logistic regression		
Coef./fixed effect	df	*χ*2	Pr(>*χ*2)	df	*χ*2	Pr(>*χ*2)
Exclosure	1	99.59	<<0.001***	1	88.81	<<0.001***
Habitat	1	3.48	0.061.	1	2.75	0.097
Species	3	41.18	<<0.001***	3	41.46	<<0.001***
Exclosure:Species	3	8.25	0.041*	3	7.65	0.054 .
Exclosure:Habitat	1	2.23	0.128	1	2.57	0.109
Habitat:Species	3	16.95	0.001**	3	17.44	0.001**

*** Significance codes: 0-0.001;

** > 0.001-0.01;

* > 0.01-0.05;

. > 0.05-0.1.

**Table 2:** Survival analyses output for multiple logistic and mixed effects logistic regression models. Variables included: ExclosureY (Protected from wild ungulate herbivory), WM (Wet meadow habitat type), POBAT (cottonwood), RIAU (currant), and SALIX (willow).

	Multiple logistic regression				Mixed effects logistic regression			
Coefficients or fixed effects	Estimate	Std. error	*z*-value	Pr(>|z|)	Estimate	Std. error	*z*-value	Pr(>|z|)
ExclosureY	1.92	0.33	5.83	<<0.001***	1.84	0.33	5.53	<<0.001***
HabitatWM	−0.97	0.39	−2.51	0.012*	−1.04	0.39	−2.65	0.008**
POBAT	−1.23	0.34	−3.59	<<0.001***	−1.33	0.35	−3.75	<<0.001***
RIAU	0.26	0.41	0.65	0.514	0.24	0.41	0.58	0.560
SALIX	−0.67	0.24	−2.82	0.005**	−0.71	0.24	−2.94	0.003**
ExclosureY:POBAT	−0.32	0.48	−0.66	0.510	−0.28	0.49	−0.58	0.561
ExclosureY:RIAU	−0.17	0.88	−0.19	0.849	−0.04	0.88	−0.05	0.963
ExclosureY:SALIX	−1.00	0.38	−2.65	0.008**	−0.95	0.38	−2.51	0.012*
ExclosureY:WM	0.61	0.41	1.48	0.138	0.65	0.41	1.56	0.119
WM:POBAT	0.48	0.59	0.82	0.415	0.55	0.59	0.93	0.352
WM: RIAU	1.52	1.21	1.26	0.209	1.59	1.22	1.31	0.192
WM: SALIX	1.61	0.42	3.84	<<0.00***	1.66	0.42	3.92	<<0.001***

*** Significance codes: 0-0.001;

** > 0.001-0.01;

* > 0.01-0.05.

**Table 3:** Likelihood ratio tests of significance for main effects from growth analysis using linear mixed effects regression.

Main effects	df	χ2	Pr( > χ2)
Exclosure	1	218.17	<<0.001***
Habitat	1	0.46	0.496
Species	3	9.76	0.021*
Height initial	1	47.20	<<0.001***

*** Significance codes: 0-0.001;

* > 0.01-0.05.

**Table 4:** Growth analyses output from linear mixed effects regression. Data from two models include: (1) initial planting height included; and (2) excluded. Variables are: ExclosureY (protected from wild ungulate herbivory), POBAT (cottonwood), RIAU (currant), SALIX (willow), and WM (wet meadow habitat type).

Fixed effects	Estimate	Std.error	*t*-value	Pr(>|t|)
Model including initial height (HeightInitial) of planting as a fixed effect				
ExclosureY	32.97	2.05	16.06	<<0.001***
POBAT	−7.27	3.23	−2.25	0.025*
RIAU	−8.56	3.47	−2.46	0.014**
SALIX	−2.75	2.15	−1.28	0.202
HabitatWM	1.74	2.45	0.71	0.478
HeightInitial	−0.39	0.06	−7.07	<<0.001***

Model excluding initial height as a fixed effect				
ExclosureY	28.70	2.03	14.14	<<0.001***
POBAT	−10.96	3.31	−3.32	0.001**
RIAU	−4.07	3.55	−1.15	0.251
SALIX	−3.54	2.23	−1.59	0.112
HabitatWM	3.14	2.52	1.25	0.213

*** Significance codes: 0-0.001;

** > 0.001-0.0;

* > 0.01-0.05.

## 2 Experimental design, materials and methods

### 2.1 Design

A stream restoration project was implemented along ~11 km of Meadow Creek, a salmonid stream in northeastern Oregon, in 2012 and 2013. During spring of 2012 and 2013, more than 50,000 native bare-root seedlings and cuttings were planted in the riparian area. Approximately equal numbers of plantings were exposed to two different herbivory treatments: (1) protected from wild ungulate herbivory using small (diameter ~2 m; height ~1.2 m) fenced exclosures; and (2) unprotected from free-ranging elk and mule deer herbivory. Protected and unprotected plantings were interspersed relatively evenly throughout the restoration area [1] in order to expose the plantings to similar abiotic and biotic conditions, and therefore, reduce the variability of other confounding variables (e.g., cold damage, drought, insect damage) between the herbivory treatments that may have impacted planting growth and survival [1]. Refer to [2] for a detailed discussion of factors that influence riparian planting survival along Meadow Creek.

191 permanently located belt transects perpendicular to the stream and extending toe-slope to toe-slope [3] were used to sample restoration plantings and naturally occurring deciduous woody vegetation. Belt transects were four meters wide, extending two meters out from the center-line on each side, and varied in length (minimum=18 m; maximum=124 m) depending on the toe-slope to toe-slope width along the stream reach. Transects were located systematically along the riparian reach with 100 m spacing, on center, along the entire reach (~11 km) and 15 m spacing, on center, in areas corresponding to a long-term manipulative experiment [1].

### 2.2 Growth and survival sampling

All plantings that occurred within a belt transect were included in sampling efforts. Sampling occurred at the beginning of growing season one (May; following the installation of plantings), at the end of growing season one (September), and at the end of growing season two (September). During each sampling period, we recorded spatial information for each planting along the transects to identify specific plantings. Spatial metrics included distance along transect, side of transect center line (upstream or downstream), and distance perpendicular to transect center line. We identified plantings to species and recorded height (cm), survival status (live/dead), exclosure use (protected/unprotected), habitat type (dry meadow/wet meadow), and evidence of browsing (yes/no). A planting was considered to be dead if no live vegetation material was observed above the soil surface with the understanding that root material may survive at or below the soil surface and have the ability to re-sprout in the future. We limited growth and survival analyses to 154 transects. 37 transects were excluded because those transects were located within larger ungulate exclosures that are part of a long-term manipulative experiment and therefore, not subject to the same herbivory treatments as the remaining 154 transects. To retain adequate sample sizes for interpretation we limited growth and survival analyses to the four most common planting species in our study area, *Salix* species (willow; survival, *n*=543; growth, *n*=293), *Crataegus douglasii* (hawthorn; survival, *n*=304; growth, *n*=226), *Populus balsamifera* (cottonwood; survival, *n*=142; growth, *n*=66), and *Ribes aureum* (currant; survival, *n*=68; growth, *n*=53).

### 2.3 Sampling of naturally occurring deciduous woody vegetation

At the end of growing season two, abundance (cover and presence/absence), height, and browsing intensity of naturally occurring deciduous woody species were measured along all 191 transects using the line intercept technique [4]. Line-intercept measurements were made by stretching a measuring tape along the center-line of the belt transects and pulling the tape taut along the ground. Cover along a transect for a specific woody species was determined by measuring the distance (cm) of each intersection along one side of the tape (≤2 m in height) with that particular species along the entire length of the transect. Then, the lengths of intersections for that species were summed, divided by the total length of the transect, and finally, multiplied by 100 to obtain percent cover for that species along the measured transect [4]. Height and browsing intensity were assessed for each individual woody plant that intercepted the transect tape. Browsing intensity (intensive or not) was assessed for each plant encountered based on the methods of Keigley [5]. A plant was considered intensively browsed if past browsing resulted in the death of a complete annual growth segment [5]. Height distributions and percentage of plants intensively browsed were plotted separately for species based on deer and elk preference (Fig. 3).

### 2.4 Data analysis

Multiple logistic and mixed effects logistic regression were used for survival analyses (Table 1, Table 2).

Both mixed effects logistic and multiple logistic regression methods were explored to see if including “site” as a random effect altered the results in an ecologically meaningful way. In both cases, survival (binary response), at the end of growing season two, was the response variable and exclosure use, habitat type, planting species, and all two-way interactions were explanatory variables. Significance of the main effects and two-way interactions were determined using likelihood ratio tests. For each effect tested, a reduced model (only the effect of interest removed) was compared to the full model. Growth data were analyzed using linear mixed effects regression. Growth (height final – height initial; measured in cm) after two growing seasons was the response variable. Site (groupings of transects for each ~1-km increment along the restored stream reach) was used as a random effect for mixed effects regression analyses. We grouped transects into a “Site” category to ensure that there were multiple observations for each level of the random effect. The explanatory variables were exclosure use, habitat type, planting species, and initial planting height (measured in May of year one concurrent with planting installation). Only major effects were included in the growth model because a likelihood ratio test gave little evidence (*p*=0.12) for the inclusion of two way interaction terms. Models with and without initial planting height were run (Table 3, Table 4). Likelihood ratio tests comparing a reduced model (removing the effect of interest) to the full model were used to test for significance of main effects (Table 3). All data analyses were performed using the statistical software R [6]. Refer to [1] for more details and specific R packages used.

## Supporting material

Supplementary file: MeadowCreekPlantingData.xlsx provides the raw dataset used for: (1) survival analysis (worksheet, ‘Survival’); (2) growth analysis (worksheet, ‘Growth’); (3) abundance and height distributions of naturally occurring deciduous woody vegetation (worksheet, ‘Nat_Occ_Species’); and (4) counts of plantings by species for each riparian transect (worksheet, ‘Planting_Density’).
