# Supplementary material for: Data and analyses of woody restoration planting survival and growth as a function of wild ungulate herbivory
Source: Data Brief. 2017 Jul 8;14:168–74. doi: 10.1016/j.dib.2017.07.002 (PMC5537423; doi:10.1016/j.dib.2017.07.002)
Supplement: Supplementary file 1 — Supplementary material [file mmc1.pdf]

Conflict of Interest statement:

I declare that no conflicting interests (financial/personal interests) or otherwise have influenced the objectivity of this research or the data generated in this study.

Sincerely,

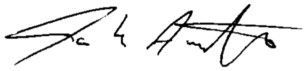

Josh Averett
