# Supplementary material for: Data and analyses of woody restoration planting survival and growth as a function of wild ungulate herbivory
Source: Data Brief. 2017 Jul 8;14:168–74. doi: 10.1016/j.dib.2017.07.002 (PMC5537423; doi:10.1016/j.dib.2017.07.002)
Supplement: Supplementary file 3 — Supplementary material [file mmc3.docx]

**Title:** Data and analyses of woody restoration planting survival and growth as a function of wild ungulate herbivory, supplementary datasets

**File name:** MeadowCreekPlantingData.xlsx

**List of data:**

1. Restoration planting growth data (worksheet; ‘Growth’)
2. Restoration planting survival data (worksheet; ‘Survival’)
3. Abundance and height distributions of naturally occurring deciduous woody vegetation (worksheet; ‘Nat_Occ_Species’)
4. Counts of restoration plantings by species along the restored stream reach (worksheet; ‘Planting_Density’)
5. Species list (worksheet; ‘Species_List)
6. **Growth worksheet**

Dimensions: 639 rows by 10 columns; rows are individual plantings, and columns are attributes

Attribute list

Index – Planting number, represents a unique individual for this worksheet.

Species – species symbol according to USDA Plants Database (<https://plants.usda.gov/java/>). See ‘Species_List’ worksheet for scientific and common names.

Exclosure Use – No = exposed to; Yes = excluded from wild ungulate herbivory

Habitat type – DM = Dry Meadow (grasses dominant); WM = Wet Meadow (sedges, rushes, or bulrushes dominant).

InitialHeight (cm) – Height to tallest live vegetation material at time (May) of planting installation

EndofYearOneHeight (cm) – Height to tallest live vegetation material at the end (September) of growing season one.

EndofYearTwoHeight (cm) – Height to tallest live vegetation material at the end (September) of the second growing season.

BROWSED(Yes/NO) – NO = no evidence of browsing; Yes = evidence of browsing at the end of the second growing season.

Growth (cm) – Difference in height to the tallest live vegetation material between the end of the second growing season and the initial height.

Site – the stream segment (~1km grouping of transects along the stream channel). Site numbers increase as you move west along the stream reach.

1. **Survival worksheet**

Dimensions: 1058 rows by 7 columns; rows are individual plantings, and columns are attributes

Attribute list

Index – Planting number, represents a unique individual for this worksheet

Species – species symbol according to USDA Plants Database (<https://plants.usda.gov/java/>). See ‘Species_List’ worksheet for scientific and common names.

Exclosure Use –- No = exposed to; Yes = excluded from wild ungulate herbivory

Habitat type – DM = Dry Meadow (grasses dominant); WM = Wet Meadow (sedges, rushes, or bulrushes dominant).

Survival_1 – 1 = planting was alive; 0 = planting was dead at the end (September) of the first growing season.

Survival_2 –- 1 = planting was alive; 0 = planting was dead at the end (September) of the second growing season.

re_sprout – planting was identified as dead (no live vegetation material above the soil surface) at the end of growing season one, but re-sprouted from the root crown and was identified as alive at the end of the second growing season.

1. **Nat_Occ_Species worksheet**

Dimensions: 1109 rows by 6 columns; rows are naturally occurring (not planted) species, and columns are attributes

Attribute list

Transect – transect number

Species - species symbol according to USDA Plants Database (https://plants.usda.gov/java/). See ‘Species_List’ worksheet for scientific and common names.

cover (%) – the measured cover using line intercept protocol for each species along a transect two years post-restoration.

group (%) – ShrubTree = species that are able to escape heavy browsing pressure in this system because they can grow above (> 50% of photosynthetic material) the browse line (~ 2.5 m in this system); Subshrubs = species that cannot grow tall enough to escape heavy browsing pressure in this system.

height (cm) – height to the tallest live vegetation material two years post restoration.

preferred – species considered as highly preferred (YES) or not (NO) by elk and deer in this system.

1. **Planting_Density**

Dimensions: 192 rows by 20 columns; rows are transects, and columns are spatial coordinates and species.

Column list

Transect – Transect number

UTMx – Universal Transverse Mercator x-coordinate

UTMy – Universal Transverse Mercator y-coordinate

Columns 4-20 are species symbols and cells contain counts of the identified species in the corresponding transect. See ‘Species_List’ for identification of species scientific and common names.

1. **Species_List**

Dimensions: 38 rows by 3 columns; rows are species, and columns are attributes.

Species Code – species symbol according to USDA Plants Database (https://plants.usda.gov/java/).

Scientific Name – Species scientific name (https://plants.usda.gov/java/).

Common Name – Species common name (https://plants.usda.gov/java/).
